# Financial investment risk prediction under the application of information interaction Firefly Algorithm combined with Graph Convolutional Network

**DOI:** 10.1371/journal.pone.0291510

**Published:** 2023-09-12

**Authors:** Muyang Li

**Affiliations:** Business school, University of New South Wales, Kensington, Sydney, Australia; University of California Los Angeles, UNITED STATES

## Abstract

This paper improves the performance of the model by Graph Convolutional Network (GCN) and Firefly Algorithm (FA) to optimize the financial investment risk prediction model. It studies the application of GCN in financial investment risk prediction model and elaborates on the role of FA in the model. To further improve the accuracy of the prediction model, this paper optimizes and improves the FA and verifies the effectiveness of the optimized model through experiments. Experimental results show that the optimized model performs well in feature selection, and the optimal accuracy of feature selection reaches 91.9%, which is much higher than that of traditional models. Meanwhile, in the analysis of the number of iterations of the model, the performance of the optimized algorithm gradually tends to be stable. When the number of iterations is 30, the optimal value is found. In the simulation experiment, when an unexpected accident occurs, the prediction accuracy of the model decreases, but the prediction performance of the optimized algorithm proposed here is significantly higher than that of the traditional model. In conclusion, the optimized model has high accuracy and reliability in financial investment risk prediction, which provides strong support for financial investment decision-making. This paper has certain reference significance for the optimization of financial investment risk prediction model.

## Introduction

With the continuous development and improvement of financial markets, financial investment risk has become one of the important issues faced by investors and financial institutions [1]. How to accurately predict financial investment risks is one of the current hot spots in financial research. Traditional risk prediction methods are often limited by the amount and quality of data, and the predictive ability of models also has great limitations [2]. In recent years, with the continuous development of artificial intelligence and Machine Learning (ML) technologies, more and more researchers have begun to apply these technologies to the financial field to improve the precision and accuracy of financial investment risk prediction [3]. Firefly Algorithms (FAs) and Graph Convolutional Networks (GCNs) are currently popular ML techniques, and they have achieved good results in various fields [4].

For the study of financial investment risk prediction, Keppo et al. (2021) conducted research and analysis on mature technologies involving big data applications in the Internet industry. Eventually, they decided to use Pandas and NumPy for the initial reading and processing of the data to clean and sort out the original data. For multi-dimensional data integration, Term Frequency–Inverse Document Frequency method was selected to extract features of customer download data. After extraction, the data was combined with customer basic attribute data to complete the data integration work. Additionally, for the problem of overfitting in the prediction of unbalanced data sets of loan default, a weighted treatment of ordinary penalty linear regression algorithm was proposed. Comparative experimental verification was conducted for four combinations of different penalty coefficients and whether they were weighted [5]. In constructing networks, Fiedler et al. (2021) directly used the physical distance of the data, the Euclidean distance, to express the degree of similarity between data sets, which was more intuitive. In terms of research implementation, firstly, the Euclidean method was used to calculate the similarity matrix of volatility between banks. A global coupling network was constructed, and hierarchical clustering analysis was used to group and classify the data. Secondly, based on the global coupling network, the Kruskal least spanning tree algorithm was used to extract the minimum path connecting 16 banks in the network. Then, the minimum path was defined as the path of risk propagation. Based on this, the degree distribution and clustering degree of the network were obtained. Finally, a simulation experiment on risk propagation was carried out using the model in the constructed least-path network [6]. Ampomah et al. (2020) systematically sorted out and summarized the research status of financial risk prediction. In view of the problems and shortcomings in the existing research, a financial risk prediction method based on weighted fusion adaptive stochastic subspace and a financial risk prediction method based on two-stage adaptive fusion stochastic subspace were constructed from the perspectives of traditional enterprise financial risk prediction and individual-oriented financial risk prediction. Then, the proposed method was verified using the data related to listed companies and the data related to personal lending in the lending market [7].

Based on traditional research, this paper studies the application of GCN in financial investment risk prediction model. The FA is optimized and promoted to improve the accuracy of the model and verify the reliability and effectiveness of the model through experiments.

## Materials and methods

### Application of GCN in financial investment risk prediction model

**Fig 1:**
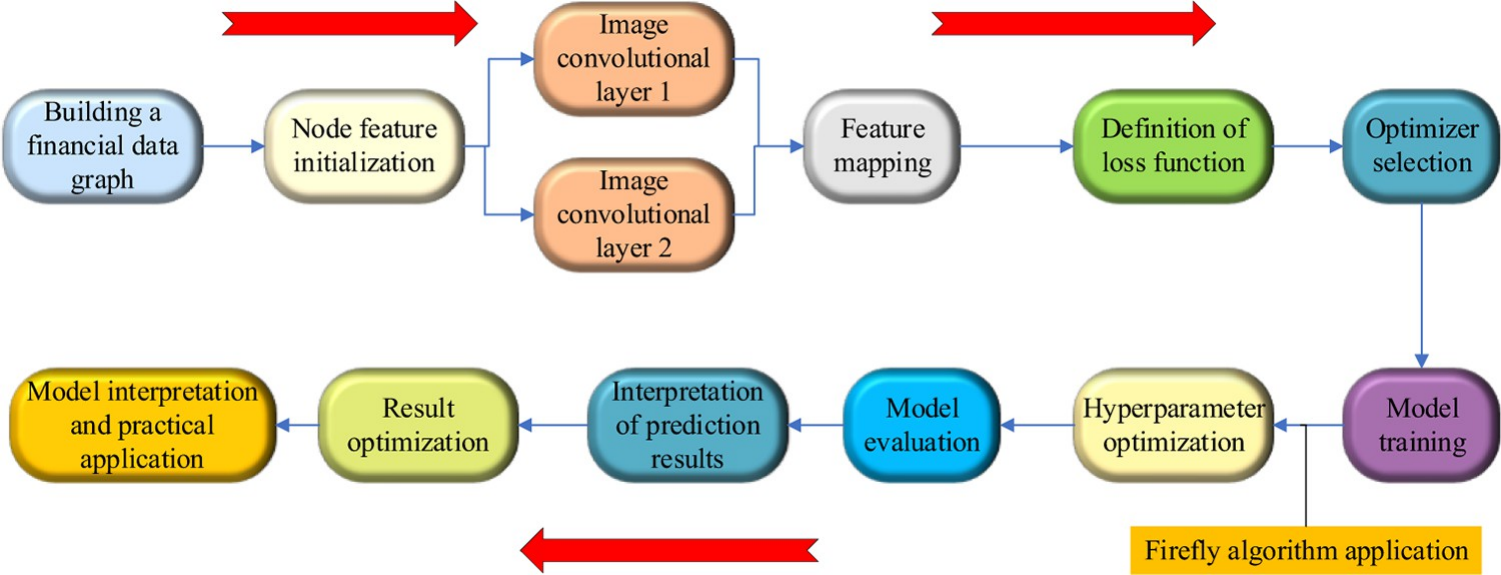
The specific process of learning graphs.

This paper combines GCN with FA to optimize the financial venture capital prediction model and improve the accuracy of prediction. GCN was originally proposed by Thomas Kipf and Max Welling in 2016 [8]. It is a semi-supervised learning method that learns the representation of nodes by performing convolution operations on the graph [9]. It can provide strong technical support for solving the prediction problem of time series. GCN utilizes the neighbor information of nodes to update node representations layer by layer by transmitting and aggregating neighbor features [10, 11]. Unlike traditional ML methods, graph learning methods represent data as a set of nodes and edges, with the relationship between data as the focus of learning [12]. This method effectively utilizes the information of graph structure and can learn node representations with better generalization performance [13]. Useful information can be extracted from the graph for effective analysis and application of the data by analyzing, clustering, classifying, and predicting nodes and edges in the graph [14]. Its specific process is shown in Fig 1.

The learning process of a graph can be expressed as:

Γ=Ω+λΦ (1)

In Eq (1), **Ω** represents the error between the predicted and true values for a particular task. ***λ*** is the hyperparameter, *Φ* is a graph regularization term used for smoothing predictions on the graph, and ***Γ*** represents the prediction result. The regularization term often implements the smoothing assumption that similar vertices tend to have similar predictions.


$$\Phi =\sum_{i=1}^{N}\sum_{j=1}^{N}g(x_{i},x_{j}){∥\frac{f(x_{i})}{\sqrt{D_{ii}}}-\frac{f(x_{j})}{\sqrt{D_{jj}}}∥}^{2}$$ (2)

**Fig 2:**
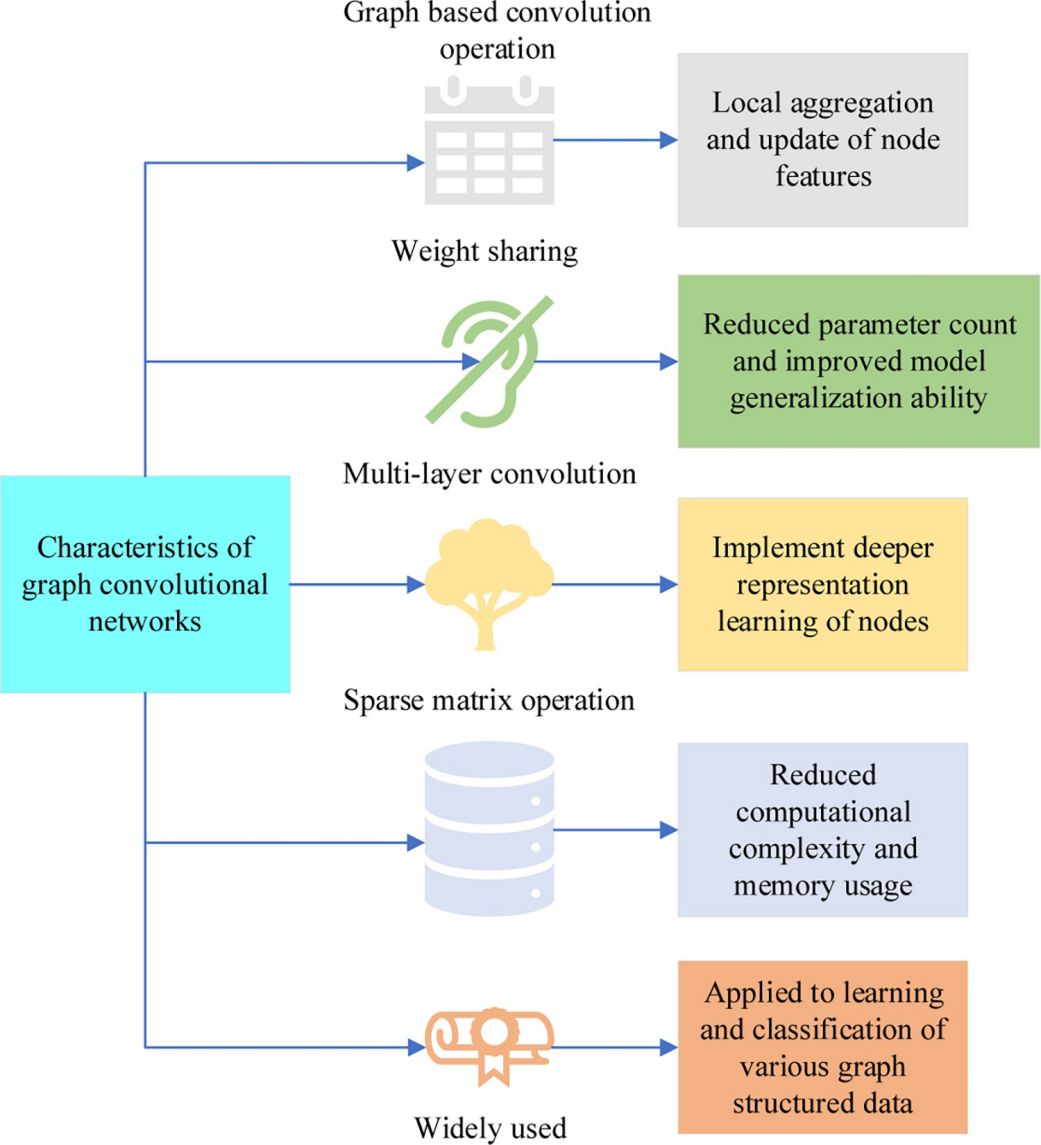
Characteristics of GCNs.

In Eq (2), ***g***(***x***_***i***_, ***x***_***j***_) represents the similarity between the feature vectors of pairs of entities. *D* is the degree of the vertex, *j* and *i* are the coordinates of the vertex, and *N* is the natural number. The regularization term smooths each pair of entities so that their predictions are close to each other [15]. GCNs can perform convolution operations on the characteristics of nodes, thereby enabling effective modeling of relationships between nodes [16]. Its main features are shown in Fig 2.

According to the idea of advanced convolutional neural networks, the purpose of GCNs is to capture local connection patterns on graphs [17]. However, the solution of applying convolution operations directly on the adjacency matrix of the graph is not feasible. When the row elements of the adjacency matrix are exchanged, the pre-exchange and post-exchange represent the same graph, but the output of the convolution changes at this time [18]. Another solution is to use spectral convolution to capture local connections in the Fourier domain, as shown in Eq (3).


$$f(F,X)=UFU^{T}X$$ (3)

**Fig 3:**
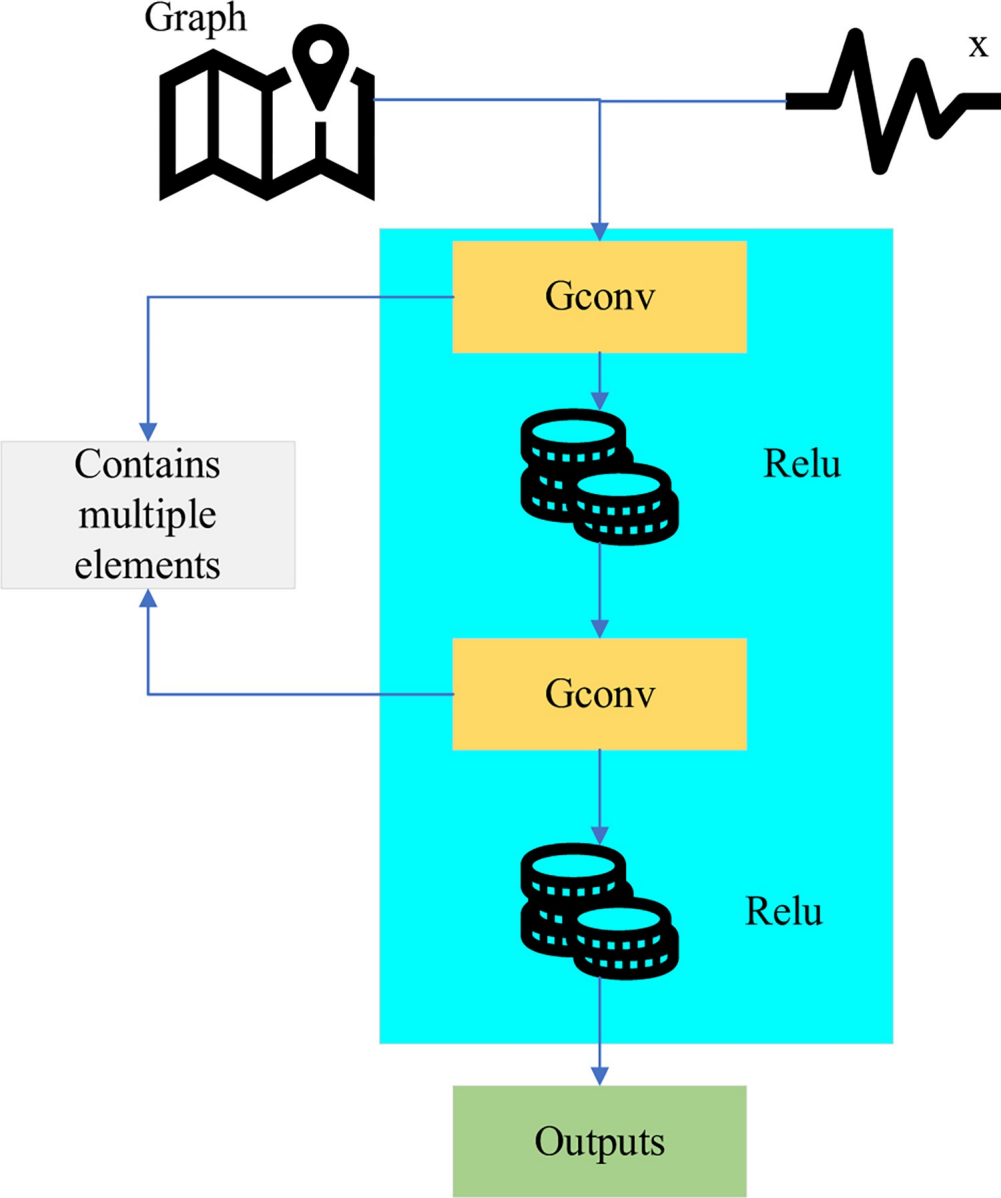
GCN structure.

In Eq (3), *f* represents the filtering operation, and *F* represents the diagonal matrix. *U* represents the eigenvector matrix of the Laplacian matrix, *X* is the image data, and *T* is the number of matrix transformations. The model structure of the GCN is displayed in Fig 3.

### The role of FA in financial investment risk prediction model

**Fig 4:**
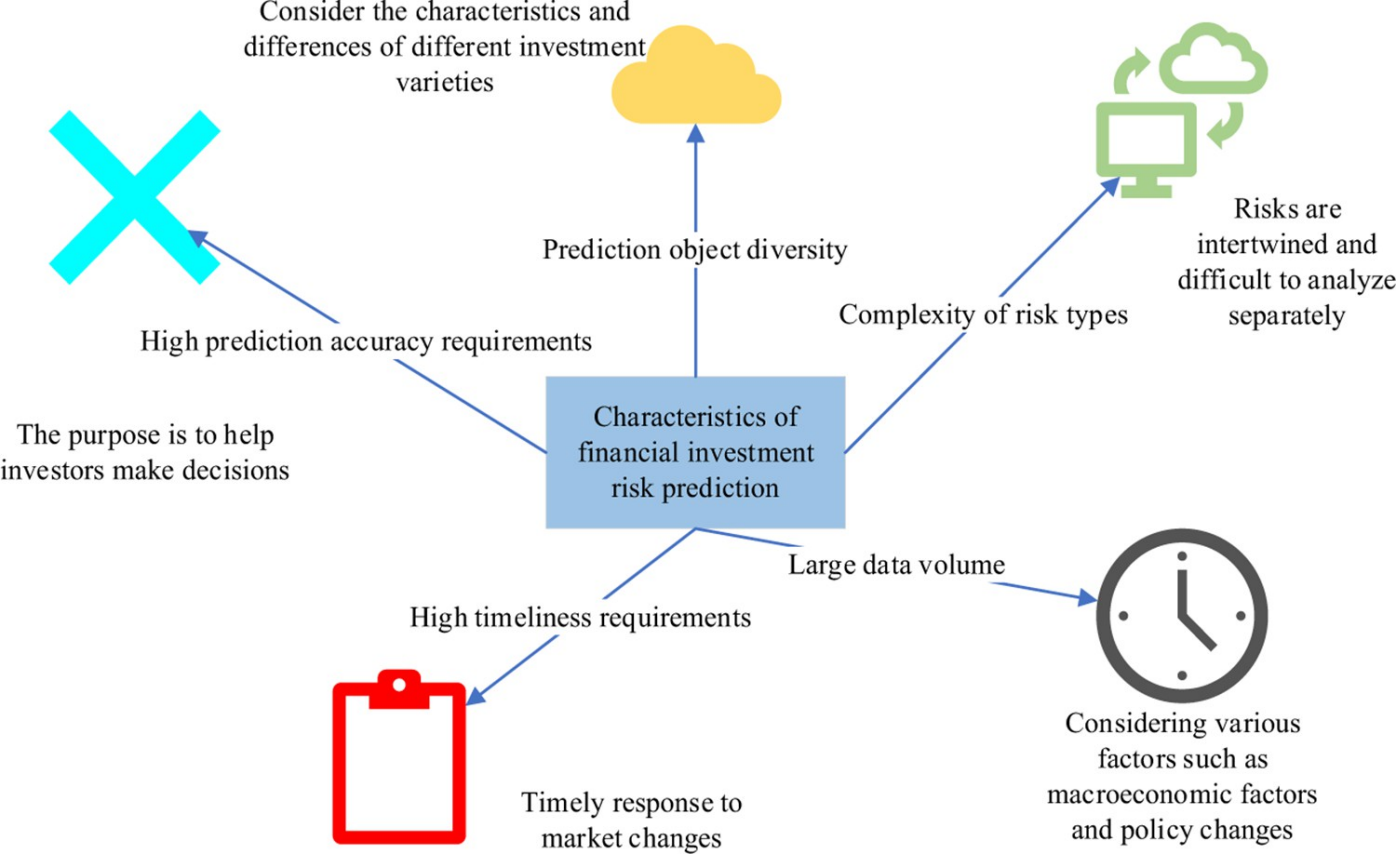
Characteristics of financial investment risk prediction.

With the massive growth of data, the complexity of data structure, and the increase of data dimensions, traditional forecasting methods are difficult to adapt to the high requirements of digital financial investment forecasting accuracy and efficiency [19]. In recent years, with the widespread application of ML in the financial field, intelligent prediction methods have been introduced into the solution of digital financial investment risk prediction problems [20]. Here, it is necessary to explain the characteristics of financial investment risk prediction, as shown in Fig 4.

The characteristics of financial investment risk prediction bring challenges to model design and algorithm selection [21]. It is necessary to perform feature selection at the data processing stage and select the most representative and differentiated features for modeling to improve the accuracy and efficiency of prediction [22]. Additionally, it is important to choose appropriate forecasting methods to meet different types of risk prediction needs. The FA is a heuristic bionic swarm intelligence algorithm that imitates the luminous behavior of fireflies in nature during courtship and foraging [23]. In the FA, each firefly represents a feasible solution to the search space, and its own brightness is positively correlated with the objective function value of its location. The higher the brightness, the better the location of the firefly [24]. The attraction of fireflies is related to their brightness, and fireflies with high brightness attract individuals with low brightness to move closer to them, thereby achieving global convergence [25]. The algorithm steps are as follows.


$$l_{i}(t)=(1-\rho )l_{i}(t-1)+\gamma J(X_{i}(t))$$ (4)

In Eq (4), ***J*** is the objective function. ***X***_***i***_(***t***) is the objective function value. ***l***_***i***_ is the fluorescein level in the iteration. t is the number of iterations. ***ρ*** is the fluorescein decay coefficient. ***γ*** is the fluorescein enhancement parameter. The fluorescein of individual fireflies decays over time, so the fluorescein value needs to be updated [26]. Then, fireflies with brightness higher than their own within the radius of the dynamic decision domain are formed into their neighborhood sets [27].


$$N_{i}(t)=\{j:∥X_{j}(t)-X_{i}(t)∥<r_{d}^{i}(t);l_{i}(t)<l_{j}(t)\}$$ (5)

In Eq (5), ***N***_***i***_(***t***) is the neighborhood set. ***X***_***j***_(***t***) is the dynamic decision domain. *j* is the function domain. $r_{d}^{i}$ is the dynamic decision domain range. The probability of an objective function value for a target individual in its neighborhood is:

$$P_{ij}(t)=\frac{l_{j}(t)-l_{i}(t)}{\sum l_{i}(t)-l_{i}(t)}$$ (6)

In Eq (6), ***P***_***ij***_ is the probability of the objective function value for the target individual within its neighborhood, and ***l***_***j***_ is the fluorescein level before iteration. Each firefly selects an individual with a higher fluorescein value than itself with a certain probability and moves towards it. Then, the equation for updating the objective function value is as follows.


$$X_{i}(t+1)=X_{i}(t)+s\times (\frac{X_{j}(t)-X_{i}(t)}{∥X_{j}(t)-X_{i}(t)∥})$$ (7)

In Eq (7), s is the moving step size, and the radius update equation of the firefly dynamic decision domain is as follows.


$$r_{d}^{i}(t+1)=min\{r_{i},max\{0,r_{d}^{i}(t)+\beta (n_{t}-|N_{i}(t)|)\}\}$$ (8)

**Fig 5:**
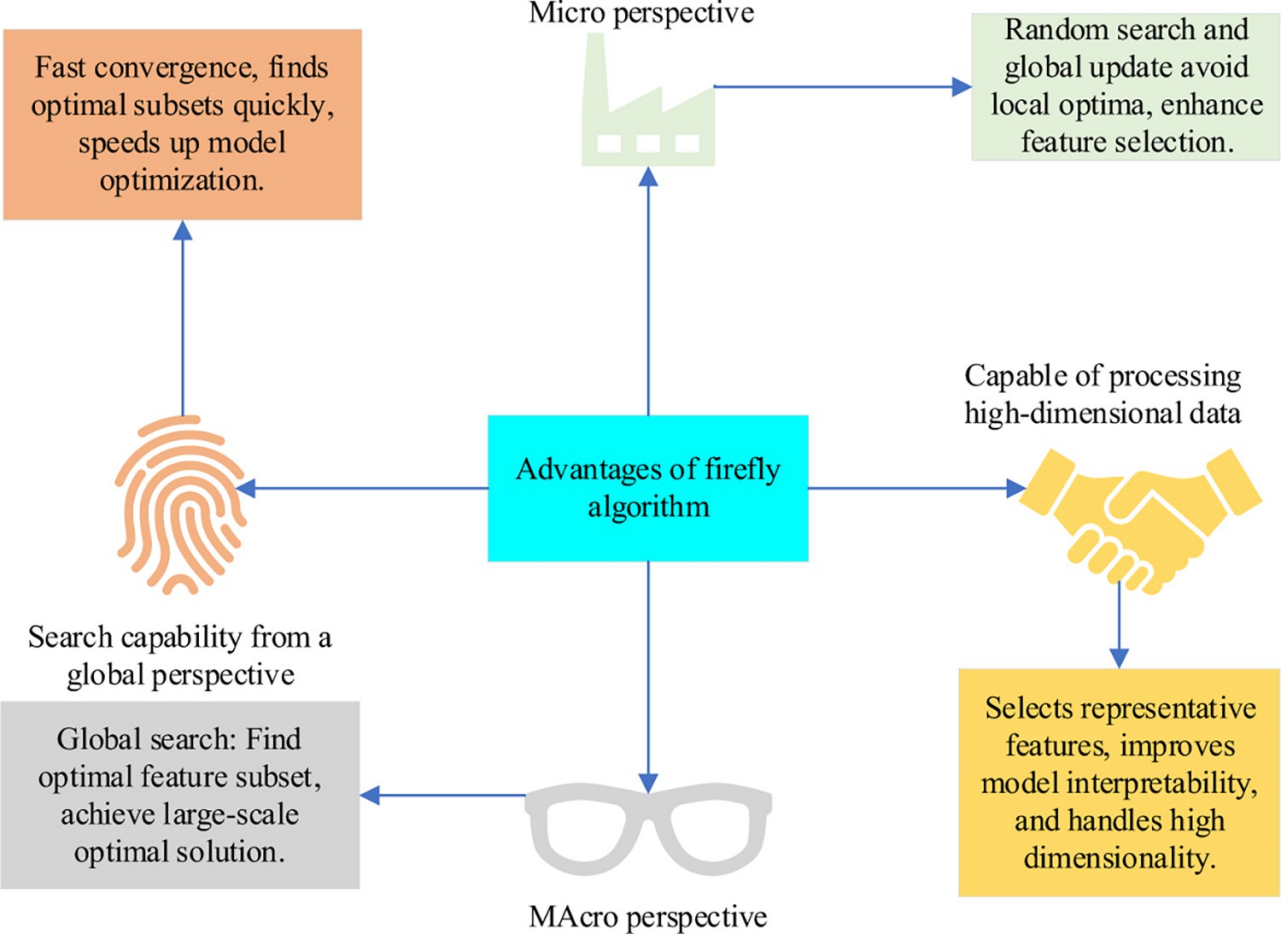
Advantages of FA.

In Eq (8), **β** is the constant coefficient, ***n***_***t***_ is the parameter that controls the number of neighbors, and ***r***_***i***_ is the perceived range of fluorescein. Each individual firefly in the search space of the FA represents a feasible solution, and its brightness is positively correlated with the objective function value of the location [28]. The higher the level of fluorescein carried by fireflies, the higher its brightness, the greater the fitness value, and the better the position. The attraction of individual fireflies is related to their brightness, and fireflies with high brightness attract individuals with lower brightness to move closer to them to achieve global convergence [29]. Compared with other heuristic algorithms, the FA has the advantages of easy implementation, strong robustness, and simple coding. It is a more effective multimodal recognition algorithm [30]. Compared with the original FA algorithm, the optimized FA has more obvious advantages in this experiment, as shown in Fig 5.

In practical application, the FA needs to adjust and optimize parameters according to specific problems to improve the search performance and result stability of the algorithm [31]. However, the FA can be combined with other theories and algorithms to achieve complementary advantages and improve the performance of the algorithm [32]. The improved mechanisms, such as population initialization, movement mode, and evolution mode, have an important impact on the performance of the FA, which also reflects that the standard FA still has shortcomings in population initialization, population diversity, and algorithm search performance [33].

### Financial investment risk prediction model based on FA and GCN

The combination of FA and GCN is realized by applying FA to the training process of GCN [34]. Specifically, the FA is used to optimize the super parameters of GCN, mainly including optimal accuracy, maximum accuracy, iterations, convergence accuracy, convergence rate, and objective function value, to maximize the model performance [35]. The goal of this combined method is to enhance the learning ability of GCNs for complex relationships between nodes in complex financial data graph structures, thereby achieving more accurate financial risk investment prediction. During the prediction process, the optimized GCN can effectively utilize the information exchange in financial data graphs and accurately predict the risks of different investment projects or assets, thereby providing scientific basis and reliable support for investment decisions [36]. The calculation method for the number of offspring produced by individual fireflies during the diffusion process is as follows.


$$N_{c}=\lfloor (D_{j}-Dmin)\times \frac{Nmax-Nmin}{Dmax-Dmin}+Nmin\rfloor$$ (9)

In Eq (9), ***N***_***c***_ is the number of offspring fireflies, and ***D***_***j***_ is the value of individual fitness. ***D*max** and ***D*min** represent the optimal and worst fitness values in the offspring, respectively. ***N*max** and ***N*min** represent the maximum and minimum values of the number of offspring, respectively. In the process of spatial diffusion, the parent generation is the axis, and the offspring are calculated according to the law of normal distribution.


$$\lambda =\lambda_{t}+(\lambda_{0}+\lambda_{t})\times \frac{{\left(\omega_{max}-\omega \right)}^{\alpha }}{\omega_{max}^{}}$$ (10)

In Eq (10), ***λ*** is the maximum standard deviation, ***λ***_**0**_ is the minimum standard deviation, ***λ***_***t***_ is the mean standard deviation, ***ω***_**max**_ is the maximum number of iterations, ***ω*** is the number of iterations, and ***α*** is the nonlinear harmonic index. When the nonlinear harmonic index is large, the convergence accuracy of the algorithm cannot be guaranteed, and the convergence speed of the algorithm is faster. When the nonlinear harmonic index is small, the convergence accuracy of the algorithm is improved, but the convergence speed is slow, and it may fall into local optimum. At this time, the tangent function needs to be introduced, because the dependent variable of the tangent function decreases with the decrease of the independent variable, and the decrease speed also shows a decreasing trend.


$$\lambda =\lambda_{i}+(\lambda_{0}-\lambda_{t})\times tan(0.875\times \frac{\omega_{max}-\omega }{\omega_{max}})$$ (11)

**Fig 6:**
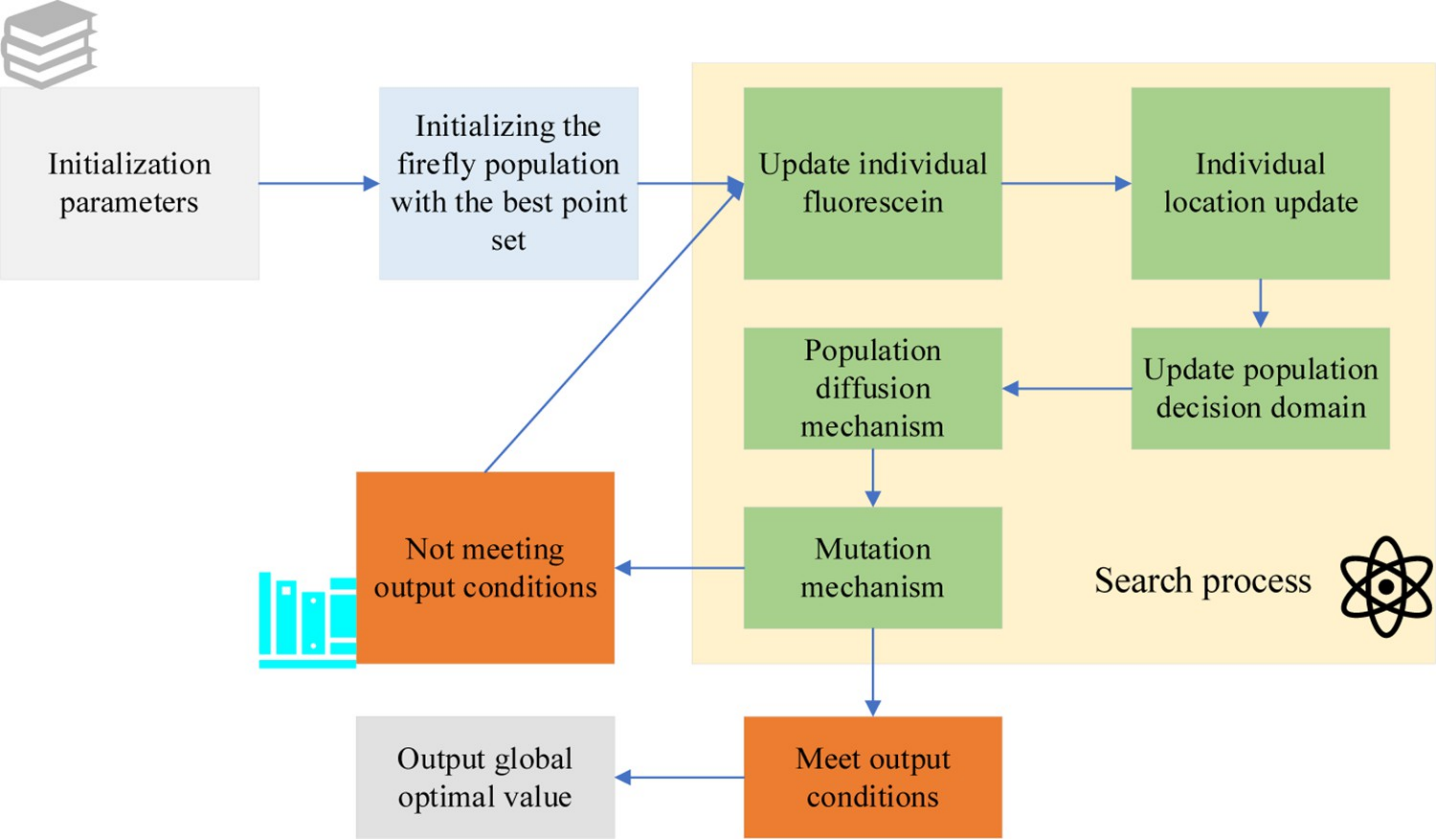
Optimized FA.

In Eq (11), **tan** is a tangent function. The attenuation rate and maximum standard deviation tend to decrease with the increase of the number of iterations of the algorithm. The larger the maximum standard deviation, the farther away the offspring are from the parent, and the stronger the algorithm’s global search ability. As algorithms evolve, the maximum standard deviation is smaller. The offspring are relatively evenly distributed around the parents, and the algorithm focuses on local search. If the optimal offspring produced are better than the parent, the current firefly is updated; otherwise, it is not updated [37]. The optimized FA is shown in Fig 6.

**Fig 7:**
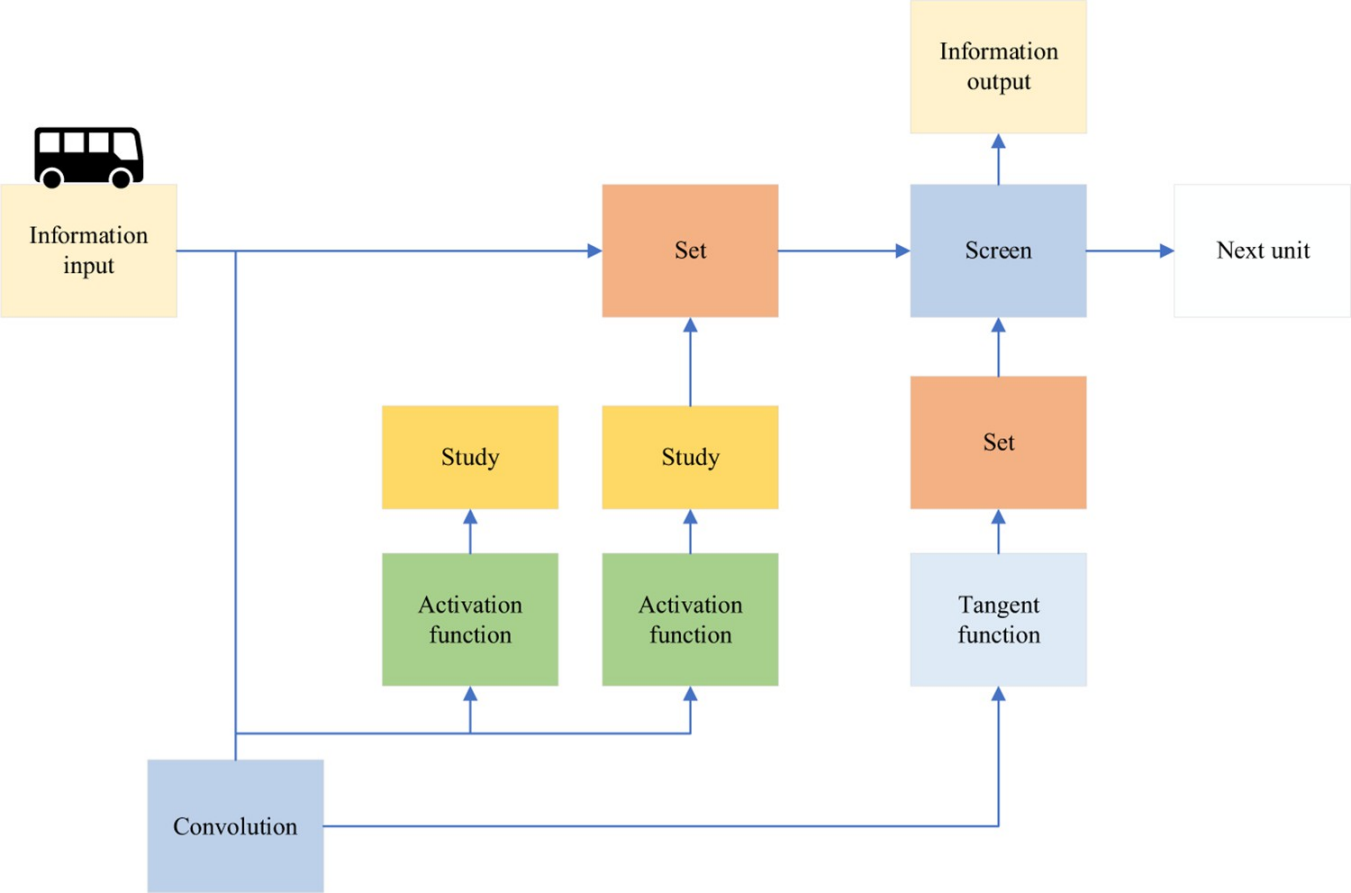
A model for integrating gated recurrent units.

The GCN can be used to learn the graph representation of financial investment and jointly predict it based on the characteristics of financial investment. The gated loop unit can be used to obtain the temporal correlation due to the continuous characteristics of the financial investment in time. The specific structure is demonstrated in Fig 7.

The update door and the reset door can be calculated as follows.


$$u_{t}=\sigma (W_{u}[\begin{array}{c} f(A,F_{t}),q_{t-1} \end{array}]+b_{u})$$ (12)

$$s_{t}=\sigma (W_{s}[f(A,F_{t}),q_{t-1}]+b_{s})$$ (13)

$${\tilde{q}}_{t}=tanh(W[f(A,F_{t}),q_{t-1}]+b)$$ (14)

$$q_{t}=(1-u_{t})*q_{t-1}+u_{t}*{\tilde{q}}_{t}$$ (15)

**Fig 8:**
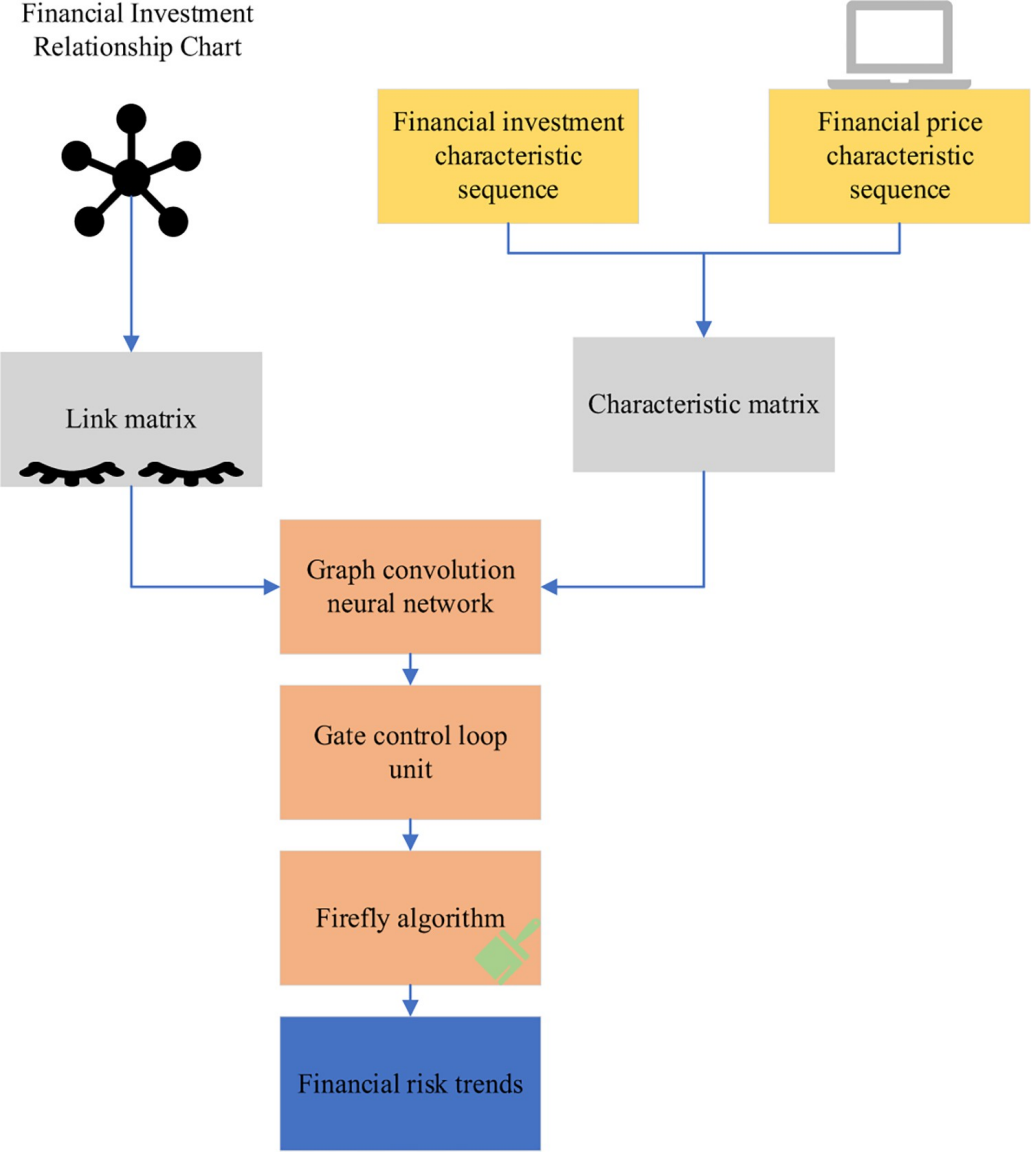
Optimized financial investment risk prediction model.

In Eq (12)–Eq (15), A is the adjacency matrix, and ***f***(***A***, ***F***_***t***_) is the graph convolution process. ***u***_***t***_ and ***s***_***t***_ are the update door and reset door, respectively. ***W***, ***W***_***u***_, and ***W***_***s***_ are all weight matrices. ***b***_***u***_, ***b***_***s***_, and b are bias values. ${\tilde{q}}_{t}$ is the output data. ***q***_***t*−1**_ is the data output of the previous unit, ***q***_***t***_ is the input data, and tanh is the hyperbolic tangent function. This model combines a GCN with a gated recurrent unit. It can process the graph and extract the correlation features of the graph and obtain the temporal correlation to complete the prediction work. Combined with the firefly optimization algorithm, the optimized financial investment prediction model is shown in Fig 8.

The model input consists of two parts, the feature matrix sequence and the adjacency matrix generated by the financial investment relationship diagram. The two parts are input into the GCN for learning representation. The relevant features of the graph structure are obtained. Then, it is input into the gated loop unit. The sequence correlation is obtained using its special gate structure. Finally, it is input into the fully connected layer and the FA to obtain the financial investment risk prediction.

### Experimental design of financial investment risk prediction model

This paper mainly studies the problem of financial risk prediction in the context of explosive visualization data. The novelty lies in the combination of FA and GCN to optimize the financial risk prediction model. Through the global search and optimization ability of the FA, the learning ability of GCN for complex financial data graphs is effectively improved. This novel fusion method brings a new perspective to financial investment risk prediction, expanding the performance and reliability of prediction models.

The experiment selects International Monetary Fund (IMF) datasets from the past three years. The IMF is one of the most important financial institutions in the world, and its dataset contains economic indicators and financial data from countries around the world. Its data sources are official institutions, such as central banks and national statistical offices of various countries. The data is compiled and processed by the IMF before being released. The experimental environment is given in Table 1.

**Table 1:** Experimental environment.

Facilities	Model
CPU (Central Processing Unit)	2.5G
Operating system	Windows7
Web	Apache-tomecat6
Memory	12G

The parameters of the model are unified to better utilize the data. The model convolutional layer is set to 2, with 64 filters per convolutional layer and a filter size of 3 * 3. The node is set to 128, the data dimension is set to 1,000, and the learning rate is 0.0001. The training period is 40, the weight attenuation is 0.0001, and the data batch size is 64. The total running time of this training is 56 hours. The equipment information and usage used are shown in Table 2.

**Table 2:** Equipment information and purpose.

Equipment name	Equipment model	Equipment quantity	Usage
Server 1	Dell PowerEdge R740	2	Model training and prediction
GPU server	NVIDIA Tesla V100	4	Deep learning model training
Workstation 1	HP ZBook Studio G7	1	Data processing and feature engineering
Workstation 2	Lenovo ThinkPad P52	1	Model optimization and evaluation

Iterative experiments are carried out by selecting different types of dataset data. The following are the eight data types involved, as shown in Table 3.

**Table 3:** IMF dataset data types.

Number	Data types	Data size	Detailed description
1	Gross Domestic Product	$10 trillion to $30 trillion	The total economic output of a country or region.
2	Inflation rate	0% to 20%	Measures the rate at which prices rise.
3	Unemployment rate	0% to 25%	The proportion of unemployed people in the workforce.
4	Trade balance	-$100 billion to $100 billion	The difference between imports and exports of a country or region.
5	Foreign exchange reserves	$100 billion to $1 trillion	The amount of foreign exchange reserves held by a country.
6	Interest rates	0.5% to 10%	The benchmark interest rate for a country or region.
7	Population	10 million to 1.5 billion	The population of a country or region.
8	Exports and Imports	0 to 200 billion	Refer to the movement of goods between countries.

The experimental dataset link matrix is a Two-Dimensional (2D) matrix. Each element represents the degree of link or connection between two data points. The actual situation of the link matrix is revealed in Table 4.

**Table 4:** Dataset link matrix.

Index	Detailed description					
Dimension	128×128 = 16,384					
Data size	128×128 = 16,384					
Example of partial content		Node 1	Node 2	Node 3	…. . .	Node 128
	Node 1	0	1	0	…. . .	1
	Node 2	1	0	1	…. . .	0
	Node 3	0	1	0	…. . .	1
	…. . .	…. . .	…. . .	…. . .	…. . .	…. . .
	Node 128	1	0	1	…. . .	0

The feature matrix of the experimental dataset is a 2D matrix. Each row represents a node, and each column represents a feature or attribute of the node. The actual situation of the feature matrix is shown in Table 5.

**Table 5:** Dataset feature matrix.

Index	Detailed description					
Dimension	128×1,000 = 128,000					
Data size	128×1,000 = 128,000					
Example of partial content		Feature 1	Feature 2	Feature 3	…. . .	Feature 1,000
	Node 1	0.2	0.8	0.5	…. . .	0.1
	Node 2	0.6	0.4	0.3	…. . .	0.9
	Node 3	0.1	0.7	0.2	…. . .	0.4
	…. . .	…. . .	…. . .	…. . .	…. . .	…. . .
	Node 128	0.9	0.3	0.6	…. . .	0.7

To better represent the performance advantages of the optimized model proposed here, the Embedded Feature Selection (EFS) model and Recursive Feature Elimination (RFE) model are selected for comparison in the experiment. RFE model conducts feature selection by recursively training a model and eliminating the least important features. Specifically, the model will first train a basic model. Then, it will calculate the impact of each feature on the model. Next, the model will delete the feature with the smallest weight and continue to train the model on the remaining features until the preset number of features is reached. EFS model embeds feature selection into model training process. Specifically, the model directly considers the importance of features during training, and feature selection is conducted according to the importance of features. Common embedded feature selection models include Lasso regression, ridge regression, and decision tree.

## Results and discussion

### Comparative analysis of ablation experimental results of three models

**Fig 9:**
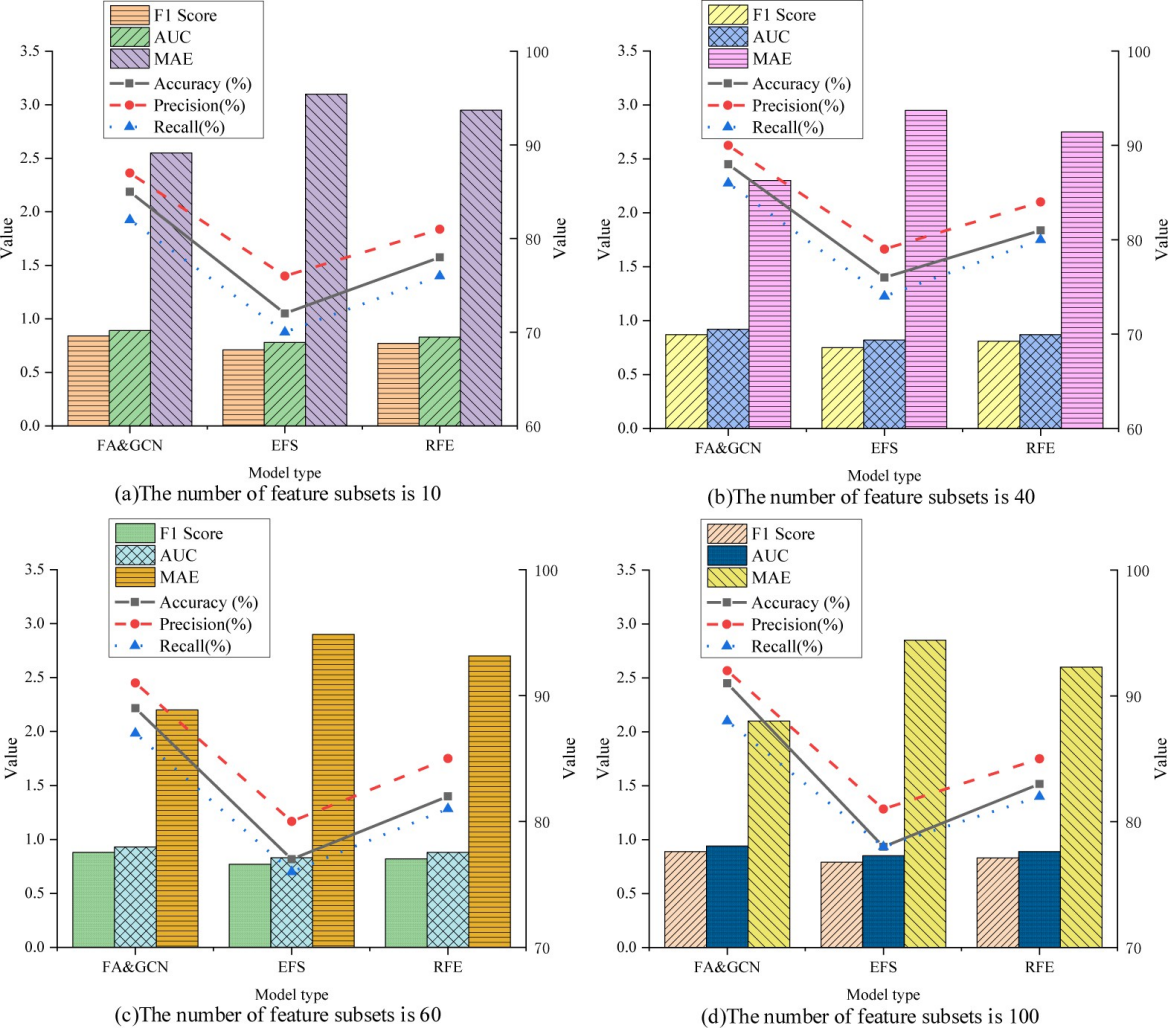
Results of ablation experiments on three models. (a) The number of feature subset is 10, (b) The number of feature subset is 40, (c) The number of feature subset is 60, (d) The number of feature subset is 40.

A dataset containing 100 features is used, where features 1 to 50 belong to category A, and features 51 to 100 belong to category B. In the ablation experiment, different numbers of feature subsets are selected and evaluated using FA&GCN, EFS, and RFE methods. Performance indicators include accuracy, precision, recall, F1 score, Area Under the Curve (AUC) and Mean Absolute Error (MAE). The ablation experimental results of three models are plotted in Fig 9.

From Fig 9, when the number of feature subsets in FA&GCN is 10, 40, 60, and 100, the accuracy is 85%, 88%, 89%, and 91%, the precision is 87%, 90%, 91%, and 92%, the recall is 82%, 86%, 87%, and 88%, the F1 scores are 0.84, 0.87, 0.88, and 0.89, the AUC is 0.89, 0.92, 0.93, and 0.94, and the MAE is 2.55, 2.30, 2.20, and 2.10, respectively. In all cases, the performance of FA&GCN is significantly better than that of EFS and RFE models. Especially when there are a large number of feature subsets, the advantages of the FA&GCN fusion model are more obvious, which supports the conclusion that the performance of the fusion model is much higher than that of the EFS and RFE models.

### Comparative analysis of financial risk prediction model feature classification performance results

**Fig 10:**
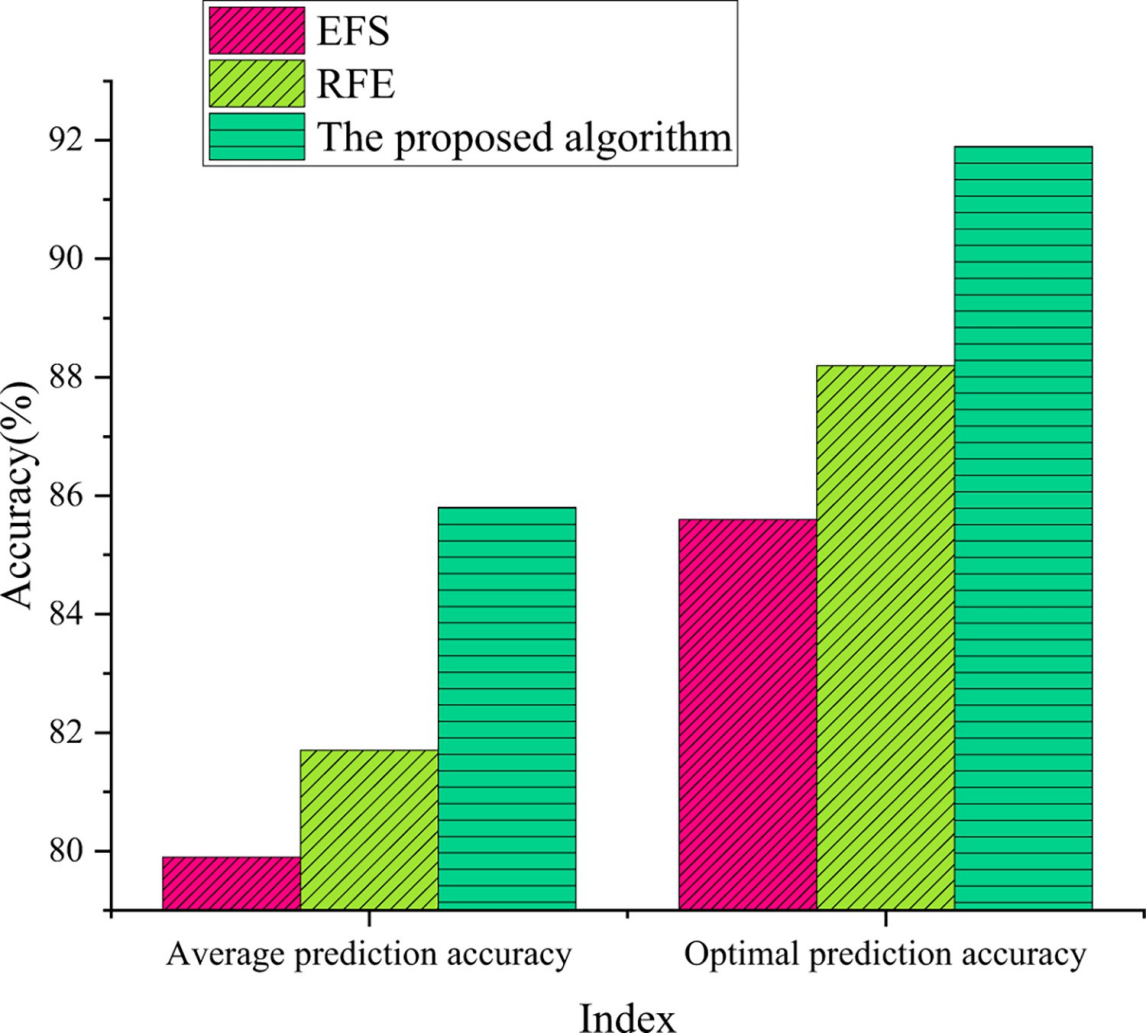
Comparison of feature selection results.

The feature classification results of the FA designed here, financial risk prediction under the GCN, EFS, and RFE models in the dataset are shown in Fig 10.

**Fig 11:**
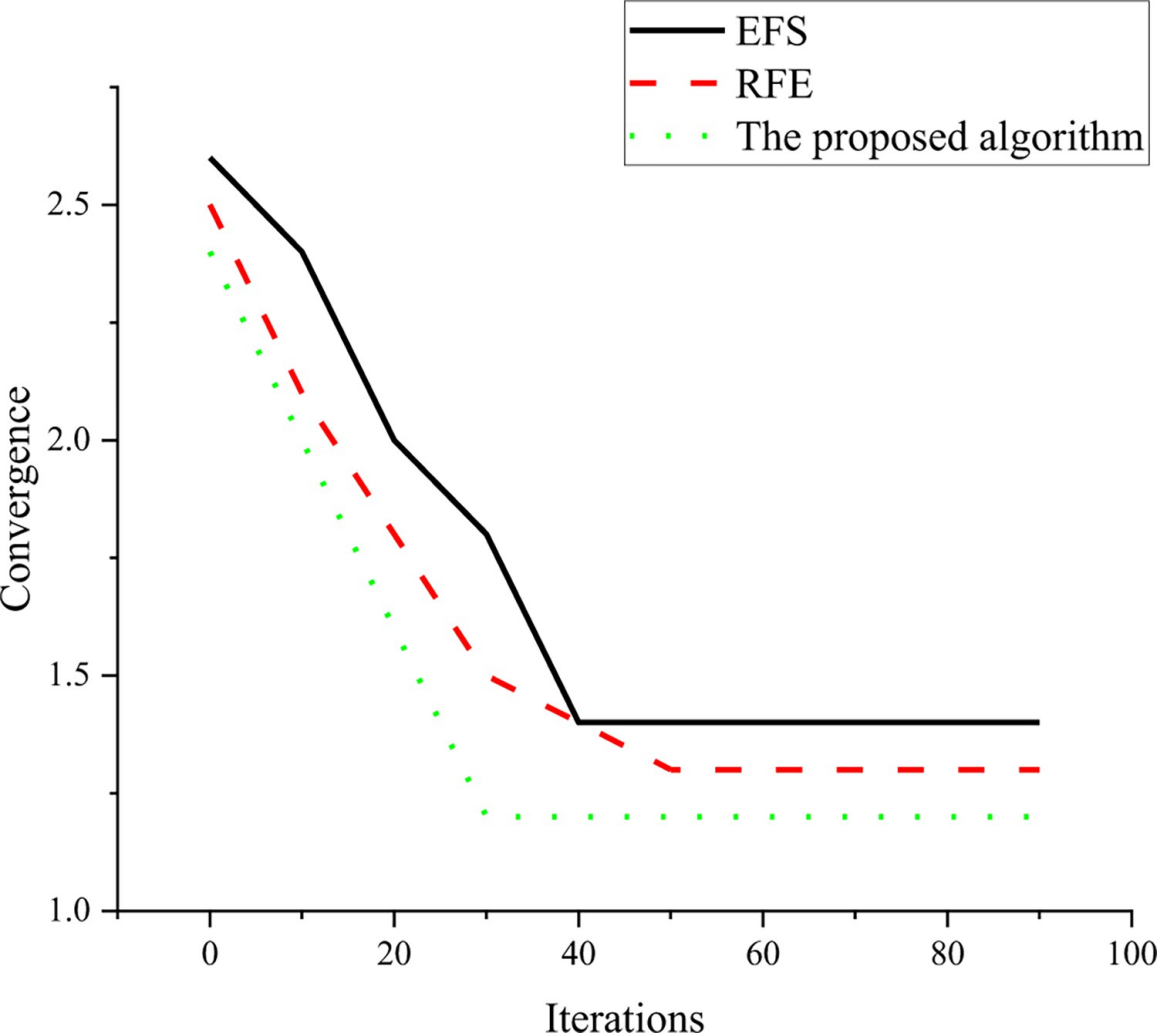
Comparison of convergence results of different models.

From Fig 10, the optimal accuracy of feature selection of the optimized model reaches 91.9%, which is much higher than that of the traditional model. This shows that the data quality of the optimized model after preliminary feature selection is better, which not only improves the accuracy of classification but also removes the influence of noise. Specifically, the optimized model improves the performance of the classifier by filtering and processing the data, removing features and noise that are not related to classification and retaining the most important features. In addition, the optimized model adopts advanced algorithms and technologies, such as RFE and L1 regularization, which further improves the accuracy and stability of feature selection and provides strong support for the accuracy and reliability of classification tasks. To explore the influence of different parameters on the model, the number of iterations of the model is increased. The convergence results of the different models are shown in Fig 11.

As shown in Fig 11, as the number of iterations increases, the objective function value of the three algorithms gradually decreases, indicating that they are all constantly optimizing the performance of the model. Besides, when the number of iterations reaches a certain value, the performance of the three algorithms gradually stabilizes and no longer fluctuates significantly. The optimized algorithm has better convergence accuracy and speed than EFS and RFE. Specifically, the optimized algorithm can achieve a smaller objective function value under the same number of iterations, indicating that it can optimize the performance of the model more effectively. The optimized algorithm also requires fewer iterations than EFS and RFE when it reaches the convergent state, indicating that it converges to the optimal solution faster. When the number of iterations is 30, the performance of the optimized algorithm tends to be stable, which means that the optimized algorithm has found the optimal solution and can stop the iteration here. In conclusion, the optimized algorithm performs well in terms of performance and convergence speed, which provides an effective method for optimizing the model.

### Comparative analysis of prediction results of financial risk prediction model

**Fig 12:**
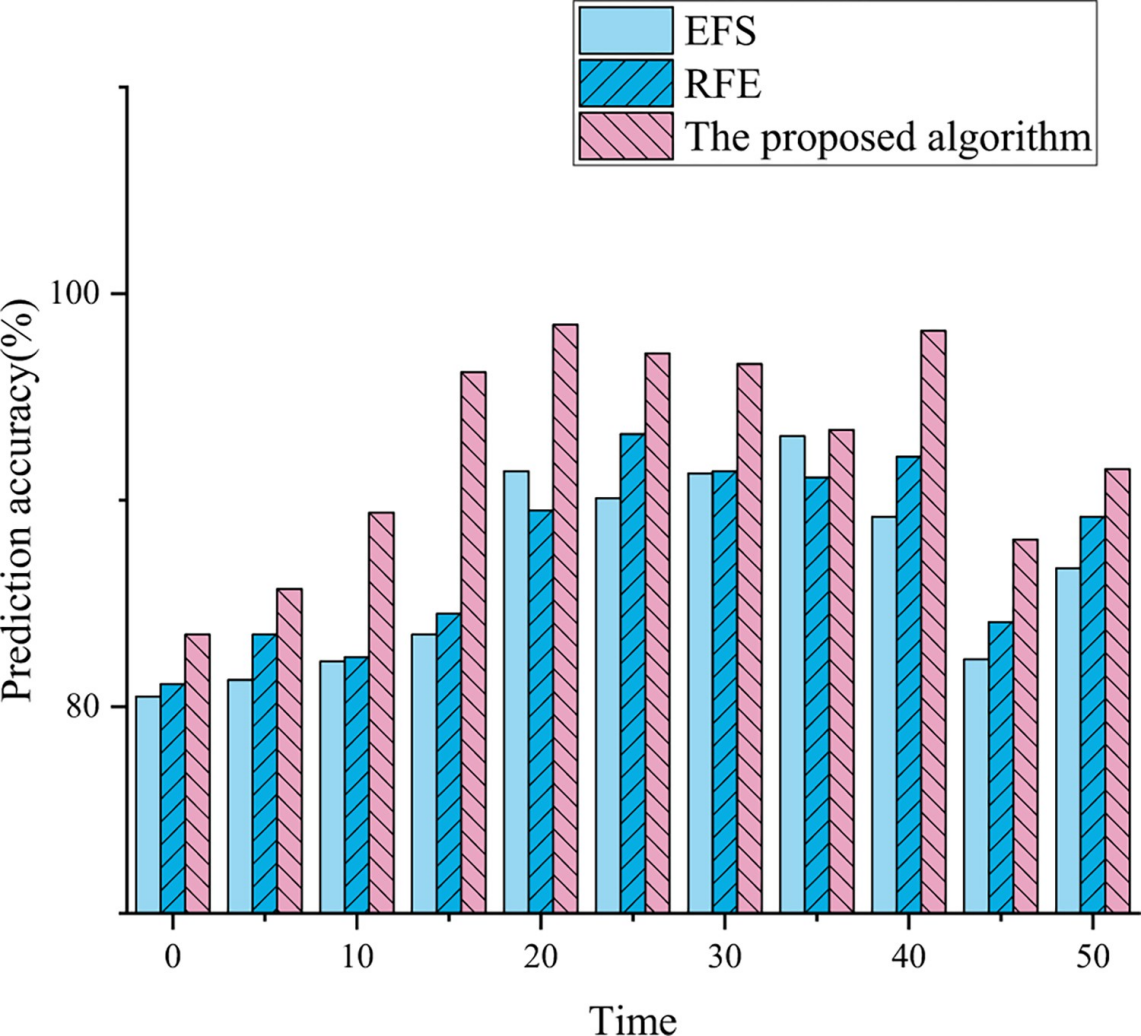
Prediction accuracy experimental results.

To verify the effectiveness of the financial feature prediction model proposed here, the experiment sets 50 timestamps and briefly closes the trading channel at the 45th timestamp. The experimental results are plotted in Fig 12.

According to the results of Fig 12, when an unexpected event occurs, the prediction accuracy of the model decreases. Although the overall curve variation trend of these three models is similar, the prediction performance of the optimized model is significantly higher than that of the traditional model. Specifically, optimized models are more stable and accurate than traditional models in terms of prediction accuracy. This may be due to the fact that the optimized model adopts the FA and the GCN, which improves the selection of model features and can better handle the noise and redundant information in the data, thereby improving the robustness and accuracy of the model.

## Conclusion

Financial investment risk has become one of the important issues faced by investors and financial institutions with the continuous development and improvement of the financial market. Therefore, this paper optimizes the financial investment risk prediction model through FA and GCN. Firstly, the application of GCN in financial investment risk prediction models is analyzed. Secondly, the role of the FA algorithm in the model is elaborated. Finally, the financial investment risk prediction model is improved by optimizing the FA algorithm, and the effectiveness of the proposed model is verified through experiments. The structure of GCN is designed, including the number of hidden layer nodes, the number of graph convolution layers, and the activation function. Appropriate loss function and optimizer are selected, and FA algorithm is used to optimize the super parameters of GCN. In the results section, the model is evaluated using ablation experimental validation and comparative validation analysis, and the accuracy, precision, recall, and other indicators of training and testing are recorded. The experimental results show that when the number of feature subsets is large, the advantages of the FA&GCN fusion model are more obvious. In feature selection, the optimal accuracy of feature selection of the optimized model in this paper reaches 91.9%, which is far higher than that of the traditional model. Besides, the number of iterations of the model is analyzed. When the number of iterations is 30, the optimal value is found, and the performance of the optimized algorithm presented here gradually stabilizes. In the simulation experiment of the financial investment risk prediction model, it is found that the prediction accuracy of the model decreases in the event of sudden accidents when the performance of the optimized algorithm is judged by controlling the timestamp. However, the prediction performance of the optimized algorithm is still significantly higher than that of traditional models.

The potential limitations and shortcomings of this paper are as follows. Firstly, the sample size and quality of the dataset may affect the generalization ability of the model. Secondly, the FA may fall into the local optimal solution in some cases, which requires more experiments and tuning. In addition, there may be differences in model performance for different types of financial data. Recognizing these details is crucial for comprehensively evaluating model performance and practicality. Especially, existing methods overlook the dynamic correlation and cyclical characteristics between financial transaction nodes. Subsequent research needs to focus on the generation of dynamic graphs and the design of multi period deep spatiotemporal prediction models to improve prediction performance.

## Supporting information

S1 File (ZIP)
