# Supplementary material for: Financial investment risk prediction under the application of information interaction Firefly Algorithm combined with Graph Convolutional Network
Source: PLoS One. 2023 Sep 12;18(9):e0291510. doi: 10.1371/journal.pone.0291510 (PMC10497170; doi:10.1371/journal.pone.0291510)
Supplement: S1 File — (ZIP) [file pone.0291510.s001.zip › 代码和数据集/Detailed description of the code.docx]

The code is mainly divided into five parts, namely, defining information interaction Firefly Algorithm (FA), defining Graph Convolutional Network (GCN) model, generating sample data and adjacency matrix, training and tuning GCN model, and comparing the test results of three models. The following is a description of the code that defines the FA for information interaction.

1. Import necessary libraries: we first imported the libraries that need to be used in the experiment, including NumPy for numerical calculations and related PyTorch libraries for defining and optimizing models.

2. Define information interaction FA class: we defined a class named FireflyAlgorithm, which was used to implement information interaction FA. In the initialization function__init__, we passed in the number of fireflies (n_fireflies), the number of iterations (n_iterations), firefly dimension (n_dim), and some algorithm parameters (alpha, beta, gamma).

3. Define fitness function: in the FireflyAlgorithm class, we defined a fitness function called fitness_function. This function is used to evaluate the fitness of fireflies, and you can define any fitness function based on the actual problem.

4. Algorithm optimization process: in the optimizing method, we executed the optimization process of the FA. Firstly, we randomly initialized n_fireflies locations of fireflies. Then, in each iteration, we had each firefly interact with all other fireflies to find a better solution. The way information is exchanged is determined by the fitness and location between fireflies. Control the intensity and randomness of information interaction by adjusting alpha, beta, and gamma parameters. Finally, we found the optimal solution position by comparing the fitness of all fireflies and returned to that position.

5. Main program: in the main program, we initialized some parameters required for the experiment, including the number of fireflies, the number of iterations, and firefly dimension. Then, we created a FireflyAlgorithm object and called its optimizing method to obtain the optimal solution. Finally, we printed out the location of the optimal solution and the corresponding fitness value.

The following is a description of the code that defines a GCN model.

1. Import the necessary library: torch: PyTorch library for constructing and training neural network models. torch.nn: the neural network module in PyTorch. torch.optim: the optimizer module in PyTorch, used to update model parameters. networkx: a library used to generate random graph data.

2. The GCN class inherits from nn.Module, indicating that it is a PyTorch model.

3. Two map convolution layers (self. gc1 and self. gc2) are initialized in the constructor. Each map convolution layer consists of a linear layer (nn.Linear) and is activated through the nn.ReLU() activation function.

4. The forward method implements the forward propagation process of the model, where x is the input feature matrix, and adj_matrix is an adjacency matrix.

5. In forward propagation, the input features are converted through two convolutional layers. Then, ReLU and softmax activation function are used to output the classification probability, respectively.

The following describes the code for generating sample data and adjacency matrix.

1. Generate sample data and adjacency matrix: generate_data function is used to generate a random graph dataset.

2. n_samples represents the number of samples, input_dim represents the input feature dimension, and output_dim represents the number of output categories.

3. X is a randomly generated feature matrix, y is a randomly generated label, adj_matrix is the adjacency matrix of the random graph generated by networkx.

4. Initialize GCN model and optimizer: create an instance model of the GCN class and specify input, hidden layer, and output dimensions.

5. Create an optimizer, use the Adam optimizer to update the model parameters, and set the learning rate to 0.01.

Train GCN model:

6. Use optimizer.zero_grad() to clear the previous gradient information.

7. Call model(X, adj_matrix) for forward propagation to obtain the predicted output.

8. Use the cross entropy loss function (nn.CrossEntropyLoss()) to calculate the loss between the prediction result output and the real label y.

9. Call loss.backward() for backpropagation and calculate the gradient of the parameters.

10. Use optimizer.step() to update model parameters.

The following is a detailed code for training and tuning the GCN model.

1. Initialize a GCN model object model and use the Adam optimizer as the optimization algorithm.

2. Set the model to evaluation mode (model.eval()) so that parameter updates do not occur during the testing phase.

3. Use with torch.no_grad() to disable gradient calculation to improve inference efficiency.

4. Use model(X, adj_matrix) for forward propagation to obtain the predicted result test_output on the test set.

5. Find the category corresponding to the maximum prediction probability of each sample using the torch.argmax function and compare it with the real label y to calculate the classification accuracy.

6. Here is a training process of 100 iterations. In each iteration, the gradient is first cleared to zero (optimizer.zero_grad()). Then, input the feature data X_train from the training set and adjacency matrix adj_matrix_train into the model to obtain the model output. Then, calculate the cross entropy loss function (nn.CrossEntropyLoss()) and carry out backpropagation (loss.backward()). Finally, update the model parameters (optimizer.step()) through the optimizer.

7. Set the model to evaluation mode (model.eval()). Then, use the test set to predict the model. Calculate predicted labels predicted_labels and calculate the accuracy based on the predicted results.

8. Simulate a new input sample x_new and the corresponding adjacency matrix adj_matrix_new, used to represent new node features and relationships between nodes. Convert the new samples into PyTorch tensors and use the optimized model for prediction. Based on the predicted results output by the model, predicted_risk is the predicted risk value, with a value of 0 or 1.

The following is a description of the code that compares the test results of the three models.

1. Test the GCN model: firstly, evaluate the GCN model by inputting the test set X_test and the corresponding adjacency matrix adj_matrix_test into the trained model.

2. Obtain the predicted result test_output. Then, use torch.argmax() to obtain the predicted label and compare the predicted result with the real label y_test and calculate accuracy accuracy_ Gcn.

3. Feature selection –Exhaustive Feature Selector (EFS) model: use the EFS method to conduct feature selection on the training set to determine the best feature combination to obtain a better feature set.

4. Then, use the selected feature selected_features_efs to slice the training and test sets to obtain the selected data X_train_selected_efs and X_test_selected_efs.

5. Feature selection –Recursive Feature Elimination (RFE) model: use the RFE method to conduct feature selection on the training set to determine the optimal number of features to retain.

6. Then, slice the training and test sets using the selected features selected_features_rfe to obtain the selected data X_train_selected_rfe and X_test_selected_rfe.

7. Initialize GCN model and optimizer (using selected features): Initialize two new GCN models model_efs and model_rfe based on the number of selected features.

8. Set the corresponding optimizers optimizer_efs and optimizer_rfe.

9. Train GCN model (using the selected features): use the selected features X_train_selected_efs and X_train_selected_rfe and the corresponding adjacency matrix adj_matrix_train to train on the new GCN model.

10. Use the same optimizer as before for parameter updates and execute n_iterations iterations of training.

11. Test on the test set (using the selected features): input the test sets X_test_selected_efs and X_test_selected_rfe and the corresponding adjacency matrix adj_matrix_test into the new GCN model to get the prediction results test_output_efs and test_output_rfe.

12. Use torch.argmax() again to obtain the predicted label.

13. Then, calculate the accuracy accuracy_gcn_efs and accuracy_gcn_rfe of the model.

14. Result printing and comparative analysis: print out the accuracy of the GCN, EFS-GCN, and RFE-GCN models for comparative analysis.
